# Supplementary material for: Patterns of Intron Gain and Loss in Fungi
Source: PLoS Biol. 2004 Nov 30;2(12):e422. doi: 10.1371/journal.pbio.0020422 (PMC532390; doi:10.1371/journal.pbio.0020422)
Supplement: Table S1 — Also available at http://genes.mit.edu/NielsenEtAl/. (4.3 MB ZIP). [file pbio.0020422.st001.zip › NielsenEtAl/html/1440.html]

AN2497.1.NCU06530.1.MG04920.1.FG04980.1


```
 CLUSTAL W (1.82) Multiple Sequence Alignments - Introns Inserted


Sequence 1: NCU06530.1	330 aa
Sequence 2: MG04920.1	332 aa
Sequence 3: FG04980.1	324 aa
Sequence 4: AN2497.1	323 aa
Alignment Length: 338 aa
Number Identitical Residues: 143 aa
Alignment Score (without introns) 7367


MG04920.1 	MSLSYYLKHLFLG-SLVTSTLARPQQKPLADDD-DDTPLPVVIWHG1LGDDYSAQGLRQV
NCU06530.1	MAISKQLKLLLTSGSLIAAALASPVQKPLNDDDIDDTPLPLVIWHG1LGDSYSAAGLREV
FG04980.1 	MLYPSLLRLGFAIGALAGHGHAAPAHT---NDDLTDTPLPVVVWHG~LGDQFNSDGMKGI
AN2497.1  	-MRPLAYTSLVALPLLTAAFPASPASS-------EFTPLPLVIWHG~LGDDYTRSGLRQV
          	   .      .    *     * *  .         ****:*:*** ***.:.  *:: :

MG04920.1 	GEIVEAANPGTFVYFIRMDDNASQDRSATFFGNLTEQVQKVCDDIASHPILSTAPAIDAL
NCU06530.1	GALADAINPGTLVYFIRMDDDGSRDRSATFLGNVTEQVAKACADIAAHPILSTAPAIDAL
FG04980.1 	EELAEAINPGTFVYIISLNGDPSQDRSATFTGNVTDQISQVCEELSKHPILSTAPAIDAI
AN2497.1  	ASLAESTNPGTYVHIIHIGDSASSDRQATFLGNVTEQLSTVCAQLAAEPILSTAPAINAL
          	  :.:: **** *::* :... * **.*** **:*:*:  .* ::: .*********:*:

MG04920.1 	GFSQGGQFLRAYVERCNSPPVRSLVTFGSQHNGIIDFKACSATDFLCKGAMALLRGNTFS
NCU06530.1	GFSQGGQFLRGYIERCNNPPVRNLVTFGSQHNGISAFRDCPWSDWVCRGAMALLKGNAWT
FG04980.1 	GISQGGQFLRGYVERCNWPQVRSLVTFGSQHNGIVKFKACGQNDWLCKGAMALLRFNVWS
AN2497.1  	GFSQGGQFLRAYVERCNNPPVRNLVTFGSQHNGIFEFQACSWGDFVCRGAEALLRVGRWS
          	*:********.*:**** * **.***********  *: *   *::*:** ***: . ::

MG04920.1 	QFVQSRLVPAQYFRDP--ANNYDKYLESSNFLADINNERALKNQTYKKNLAKLVNFVMFA
NCU06530.1	QFVQSRLVPAQYYRN---LDEYEQYLEHSNFLADINNERMFKNRTYRENLAKLENLVLYM
FG04980.1 	NFVQSRLVPAQYYRDPSTEQDYNNYLENSNFLADINNERELKNVKYKANLGSLTNFVMWM
AN2497.1  	NLVQSRFVPAQYFRDP---AELGEYLENSNFLADVNNERVLKNATYKKNLSTLNRFAMFM
          	::****:*****:*:.    :  :*** ******:**** :** .*: **..* .:.:: 

MG04920.1 	FDEDTTVIPRETAWFEEVNGT--EVIPLRARKLYTEDWIGLRELDRNGGLKLRTCPGEHM
NCU06530.1	FEDDTTVVPKETAWFEEVNGT--EITPLRARKMYSEDWIGLRELDRKGGLHFRTAPGDHM
FG04980.1 	FEDDTLVVPKESSWFEEVNGT--ESIPLRARKLYQEDWLGLRGVDRNGGLHFRSAPGDHL
AN2497.1  	FEDDTIVHPKESSWFAEVNTTTGEVTPLRERDIYKEDWLGLRVLDEKGALDFKTLSGEHM
          	*::** * *:*::** *** *:.*  *** *.:* ***:*** :*.:*.*.::: .*:*:

MG04920.1 	QLSDELLNKTFTEFFGPNNKKFGMGGQAGDLDVTREDEL
NCU06530.1	QLTDQLLNETFAEFFGPIKKAKKSAPRP--LMLGGGDL-
FG04980.1 	QGMDELLNQTFTDYFGPWKRSFSEDVWQ---EL------
AN2497.1  	QLAEEDLVEVFEKYFGPVEVDLPPAQFH---LVNQEGY-
          	*  :: * :.* .:*** :             :   .
```
